# Online Group Hypnotherapy for Irritable Bowel Syndrome—a Pilot Study

**DOI:** 10.1111/nmo.70328

**Published:** 2026-04-29

**Authors:** Jenny Lövdahl, Hans Törnblom, Gisela Ringström, Olafur S. Palsson, Magnus Simrén

**Affiliations:** ^1^ Department of Molecular and Clinical Medicine, Institute of Medicine, Sahlgrenska Academy University of Gothenburg Gothenburg Sweden; ^2^ Department of Medicine Sahlgrenska University Hospital, Gothenburg Gothenburg Sweden; ^3^ Division of Gastroenterology and Hepatology University of North Carolina at Chapel Hill Chapel Hill North Carolina USA

**Keywords:** brain–gut axis, digital health, hypnosis, irritable bowel syndrome, nurses, psychotherapy

## Abstract

**Background:**

Gut‐directed hypnotherapy is an effective treatment for patients with irritable bowel syndrome (IBS). Group delivery and nurse‐led hypnotherapy can increase availability. Online treatment shows promising results, but this has not been tested in a group format.

**Aims:**

To investigate the acceptability and efficacy of nurse‐led, online group hypnotherapy in patients with IBS.

**Methods:**

Patients received eight sessions of gut‐directed hypnotherapy in groups via live video conferencing. IBS symptoms were assessed at baseline, mid‐treatment, after treatment, and at follow‐up. Patients who reported an IBS‐SSS reduction of ≥ 50 points were considered responders. Extracolonic symptoms, psychological symptoms, and quality of life were assessed, as well as usability and treatment satisfaction. The study results were compared to previous assessments of group hypnotherapy delivered on‐site. After hypnotherapy, patients were asked which treatment modality (online or on‐site) they would prefer.

**Results:**

We included 51 patients. IBS severity was reduced after hypnotherapy (median IBS‐SSS: 304 (225–385) vs. 225 (172–312), *p* < 0.001), and 27 patients (53%) were responders. These results are comparable to on‐site group hypnotherapy outcomes; IBS‐SSS: 310 (232–368) versus 230 (151–330), *p* < 0.001, responders: 55%. Symptom reduction was sustained at six‐month follow‐up. Quality of life, extracolonic, and psychological symptoms also improved. The patient ratings of the usability of the video call platform and treatment satisfaction were high.

**Conclusions and Inferences:**

Nurse‐led, gut‐directed group hypnotherapy delivered online is acceptable, often preferred by patients, and has comparable efficacy to in‐person group hypnotherapy. By combining group and online treatment, hypnotherapy can be made more accessible for patients.

## Key Points

- Gut‐directed hypnotherapy is a brain–gut behavioral therapy that is effective for patients with irritable bowel syndrome, but access to treatment is limited.
- In this pilot study, we tested to give nurse‐led group hypnotherapy using a digital video platform to increase accessibility further.
- Study outcomes show that the treatment modality is feasible, efficacious, and acceptable for patients.

## Graphical abstract

Gut‐directed hypnotherapy is an effective brain–gut behavioral therapy for irritable bowel syndrome (IBS), but access is limited. This pilot study shows that online group hypnotherapy is feasible, acceptable, and similarly effective to hospital‐based delivery.

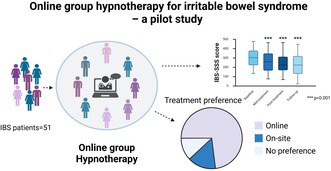

## 1 Introduction

Irritable bowel syndrome (IBS) is one of the most common disorders of gut–brain interaction (DGBI), present in 4%–9% of the global population according to recent epidemiological estimations [1, 2]. It is characterized by recurrent abdominal pain associated with changes in stool frequency, form, or appearance. In addition, abdominal bloating and distention are also frequently reported, and extracolonic symptoms, such as bodily pain and fatigue, are often present. IBS is female predominant, and the onset of the disorder is more common at a younger age [3]. IBS is commonly associated with the presence of anxiety and depression [4], and co‐occurring psychological distress is linked to more severe IBS symptoms and a poorer quality of life [5]. Brain–gut behavioral therapies, such as gut‐directed hypnotherapy, cognitive behavioral therapy, and mindfulness‐based stress reduction, play an important role in the multidisciplinary management of IBS, alongside dietary and medical interventions [6]. Gut‐directed hypnotherapy is an effective treatment and has predominantly been offered to patients who experience inadequate symptom relief from standard management, and where symptoms of anxiety and/or depression are more frequent [7]. However, the accessibility of gut‐directed hypnotherapy is poor due to the lack of suitably trained therapists and the time‐consuming nature of this treatment. Different approaches to enhance availability, like group delivery [8, 9, 10] or nurse‐led hypnotherapy using a scripted protocol [11], have shown promising results. Remote delivery could potentially increase accessibility further, enabling patients who have difficulties traveling to treatment centers to get access to hypnotherapy. Gut‐directed hypnotherapy normally includes 6–12 sessions, and the possibility to receive the treatment remotely, for instance during a longer work break if working from home or in a place where you can be undisturbed, could be a practical advantage compared to traveling to the hypnotherapy treatment center. Incapacitating symptoms or living far from treatment centers can also hinder patients from receiving hypnotherapy on‐site. Palsson et al. have reported that gut‐directed hypnotherapy delivered remotely using audio files was effective for IBS patients, although the response rates were somewhat lower compared to hypnotherapy delivered by therapists at the specialized center [12]. Audio files have also been tested as home‐based hypnotherapy for children with IBS or functional abdominal pain with long‐term effects comparable to in‐person hypnotherapy [13]. Remote non‐therapist delivery via mobile health applications using prerecorded hypnotherapy sessions has shown effectiveness [14] for those who complete the hypnotherapy program, although low adherence to the treatment has been reported to be a problem [15, 16]. Delivery by video calls has also been tested. A study evaluating individual Skype hypnotherapy for IBS patients reported it to be feasible and effective [17]. In general, telehealth solutions are becoming increasingly common, with the COVID‐19 pandemic catalyzing the implementation of digital solutions. According to an international survey conducted in 2023, 76.9% of clinicians providing gut‐directed hypnotherapy for patients with IBS had used video conferencing at some point when delivering hypnotherapy [18]. Although remote hypnotherapy is used more frequently, it is often delivered individually. By administering this treatment option in groups, accessibility could increase further. Online group hypnotherapy is used for patients with DGBI in a limited number of centers in the United States [19]. Although treatment effectiveness has not yet been reported, a qualitative study has provided valuable insights into patients' experiences of hypnotherapy [20]. Overall, the experience was positive, with 70% of patients reporting that they would have preferred the remote delivery format if given the choice. Additionally, half of the patients indicated a preference for the group format over individual treatment. The aim of this study was to assess the feasibility and efficacy of delivering online gut‐directed hypnotherapy via live video sessions in groups, and to compare the results with existing results in patients who have received in‐person group hypnotherapy at the hospital.

## 2 Methods

### 2.1 Participants

Patients who fulfilled the Rome IV criteria [3] for IBS and who had refractory IBS symptoms were consecutively recruited to the online group hypnotherapy study between 2022 and 2024. The enrollment and hypnotherapy treatment took place at a specialized combined clinical and research neurogastroenterology outpatient clinic at Sahlgrenska University Hospital, Gothenburg, Sweden. To assess if the effect on IBS symptoms with online group hypnotherapy was comparable with previous results obtained via treatment on‐site, these were compared with a group consisting of two different cohorts of IBS patients, recruited at the abovementioned outpatient clinic. The first cohort was recruited between 2011 and 2019 and received group hypnotherapy on‐site as a part of their routine clinical management, monitored within a clinical audit [11]. The other group was recruited between 2011 and 2016 and received group hypnotherapy on‐site within a randomized controlled trial comparing individual and group hypnotherapy [10]. Either the Rome III [21] or IV [3] criteria were used in the comparison group, depending on the time of recruitment. The patients had been referred to the gastroenterology unit by general practitioners or gastroenterologists. The recruitment process was conducted by two gastroenterologists (MS and HT), who confirmed the IBS diagnosis and assessed eligibility based on the inclusion and exclusion criteria (Table 1). All patients were thoroughly informed about the study and signed a written informed consent prior to participation. This study and the previously reported studies with the comparison groups were approved by The Regional Ethical Review Board in Gothenburg (diary numbers: 686‐11, 636‐12, and 2022‐03997‐01), and were registered with the ClinicalTrials.gov study protocol IDs: NCT06297785, NCT06167018, and NCT03432078.

**TABLE 1:** Inclusion and exclusion criteria.

Inclusion criteria
Signed written informed consent
2 Age 18–75 years
3 IBS according to the Rome IV criteria
4 Basic knowledge of the Swedish language
Exclusion criteria
Other diseases that could affect gastrointestinal symptoms
2 Severe psychiatric disease
3 Pregnancy
4 Recent or ongoing life crisis
5 Ongoing participation in other clinical study
6 Previous treatment of gut‐directed hypnotherapy
7 Medical treatment changes that could affect IBS symptoms during the last 3 months

### 2.2 Online Group Hypnotherapy

Patients received eight live video sessions in groups of six to eight participants, during a period of 12 weeks. The official telehealth application of the hospital was used during the sessions. Patients were instructed to participate in the video session in a room at home or elsewhere where they could be alone, undisturbed, and with their web camera turned on. Sessions were based on the North Carolina Protocol [22] and were given on a weekly basis during the first 4 weeks, and after that, every other week. A session normally lasted for about 30–40 min. In addition to the sessions, participants were instructed to use an audio file with a hypnotic exercise daily during the treatment period. The gut‐directed hypnotherapy was administered by a nurse specialized in cognitive behavioral therapy and hypnotherapy (JL). Patients in the comparison group received hypnotherapy on‐site at the hospital. The gut‐directed group hypnotherapy treatment delivered on‐site was conducted in the same manner as for the online group hypnotherapy patients, that is, patients received an equivalent number of hypnotherapy sessions at identical intervals and used the same hypnosis audio exercise between sessions.

### 2.3 Questionnaires

To assess efficacy, usability, and treatment satisfaction, patients completed the following self‐reported questionnaires: *The IBS Severity Scoring System* (*IBS‐SSS*) measures the severity of IBS symptoms and consists of five items that measure the severity and frequency of abdominal pain, the severity of abdominal bloating, bowel habit dissatisfaction, and life interference. Scores range from 0 to 500, with a higher score indicating more severe symptoms. A reduction of 50 points is considered to reflect an improvement of clinical relevance. Symptom severity can also be classified into four levels based on the total score: remission (< 75), mild (75–175), moderate (175–300), and severe (≥ 300) [23]. The questionnaire also contains an additional domain measuring extracolonic symptoms, including nausea/vomiting, early satiety, headaches, backaches, lethargy, excess wind, heartburn, urinary symptoms, thigh pain, and bodily aches, with scores ranging from 0 to 500 [24].

*The Visceral Sensitivity Index (VSI)* measures gastrointestinal (GI)‐specific anxiety. It consists of 15 different questions with six‐point Likert scale response options, with a total score range from 0 to 75, where a higher score indicates a higher severity of GI‐specific anxiety [25].

*The Hospital Anxiety and Depression Scale (HADS)* measures general anxiety and depression. It consists of two subscales with seven questions each, with response alternatives using four‐point Likert scales. Each subscale can generate a score from 0 to 21. A higher score indicates more severe anxiety or depression [26].

*The IBS Quality of Life Questionnaire (IBSQOL)* is a disease‐specific, health‐related quality of life instrument that consists of nine domains considered relevant for IBS patients: emotional health, mental health, sleep, energy, physical functioning, diet, social role, physical role, and sexual relations. The total score for each subscale can range from 0 to 100, with a higher score indicating a better health‐related quality of life [27].

*The System Usability Scale (SUS)* is a 10‐item, five‐point Likert scale questionnaire that measures the perceived usability of a system or a product, widely used to evaluate software products. A total score is calculated from the 10 items, ranging from 0 to 100. A higher score indicates greater usability. A score of 71 is considered to be the lower limit of acceptability for a system [28].

*The Client Satisfaction Questionnaire (CSQ‐8)* measures the patients' level of satisfaction of mental health services. It consists of eight items on a four‐point Likert scale, generating a maximum score of 32. A higher score indicates greater treatment satisfaction [29, 30]. A mean score of 27.09 (SD = 4.01) has been reported as an average for a variety of outpatient and inpatient mental health services provided on‐site [31].

### 2.4 Data Analysis and Statistics

**FIGURE 1:**
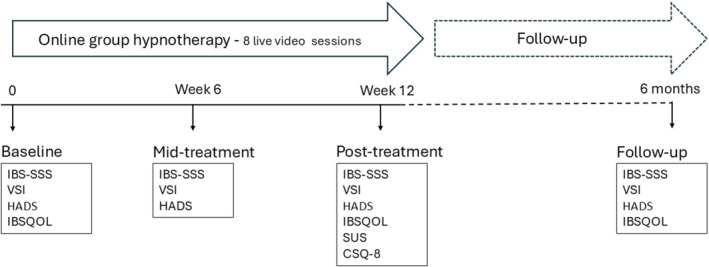
Study outline for online group hypnotherapy. IBS‐SSS, IBS severity scoring system; IBSQOL, IBS quality of life questionnaire; CSQ‐8, client satisfaction questionnaire; HADS, hospital anxiety and depression scale; SUS, system usability scale; VSI, visceral sensitivity index.

Clinical and demographic information was collected before treatment for all patients. Patient‐reported outcomes (IBS symptoms, extracolonic symptoms, psychological symptoms, and quality of life) were measured prior to, during and after the hypnotherapy program (posttreatment and at follow‐up 6 months after treatment initiation) (Figure 1). In addition to this, patients who received online group hypnotherapy assessed software usability and treatment satisfaction posttreatment. They were also asked which treatment modality they would prefer, that is, online group hypnotherapy or group hypnotherapy on‐site, if they had been able to choose, and the reasons for their preference. Participants in the online group hypnotherapy study completed the questionnaires electronically, while the comparison group, receiving group treatment on‐site, completed paper questionnaires.

The primary endpoint was improvements in IBS symptoms. A response to treatment was defined as a reduction of the IBS‐SSS score of 50 points or more relative to baseline, which is a recommended and widely used cutoff to detect clinical improvement [23]. Changes regarding proportions of patients in the IBS symptom severity groups based on IBS‐SSS (remission, mild, moderate, severe) were compared between the posttreatment and baseline assessments. The secondary endpoints were changes in extracolonic symptom severity (IBS‐SSS), GI‐specific anxiety (VSI), general anxiety (HADS‐A), depression (HADS‐D), and quality of life (IBSQOL) at the posttreatment assessments compared with baseline, as well as software usability (SUS) and treatment satisfaction (CSQ‐8). Intention‐to‐treat (ITT) analyses were used for IBS symptoms. The last‐observation‐carried‐forward principle was used to impute missing values for dropouts and participants who completed the hypnotherapy program but missed assessing symptoms posttreatment. No formal statistical comparisons were made between the online group hypnotherapy patients and the comparison group of patients previously treated with group hypnotherapy on‐site. Secondary outcomes (extracolonic symptoms, psychological symptoms, and quality of life) were analyzed using Per‐Protocol (PP) analysis. Wilcoxon signed‐rank tests were performed to evaluate changes in all symptoms assessed following hypnotherapy. Results for continuous variables are shown as median and interquartile range (IQR), and categorical variables as proportions (percentages). The conventional 0.05 level was used as the cutoff for statistical significance. All statistical analyses were performed with the IBM SPSS Statistics program, version 29.0.0.0.

## 3 Results

### 3.1 Participants

**FIGURE 2:**
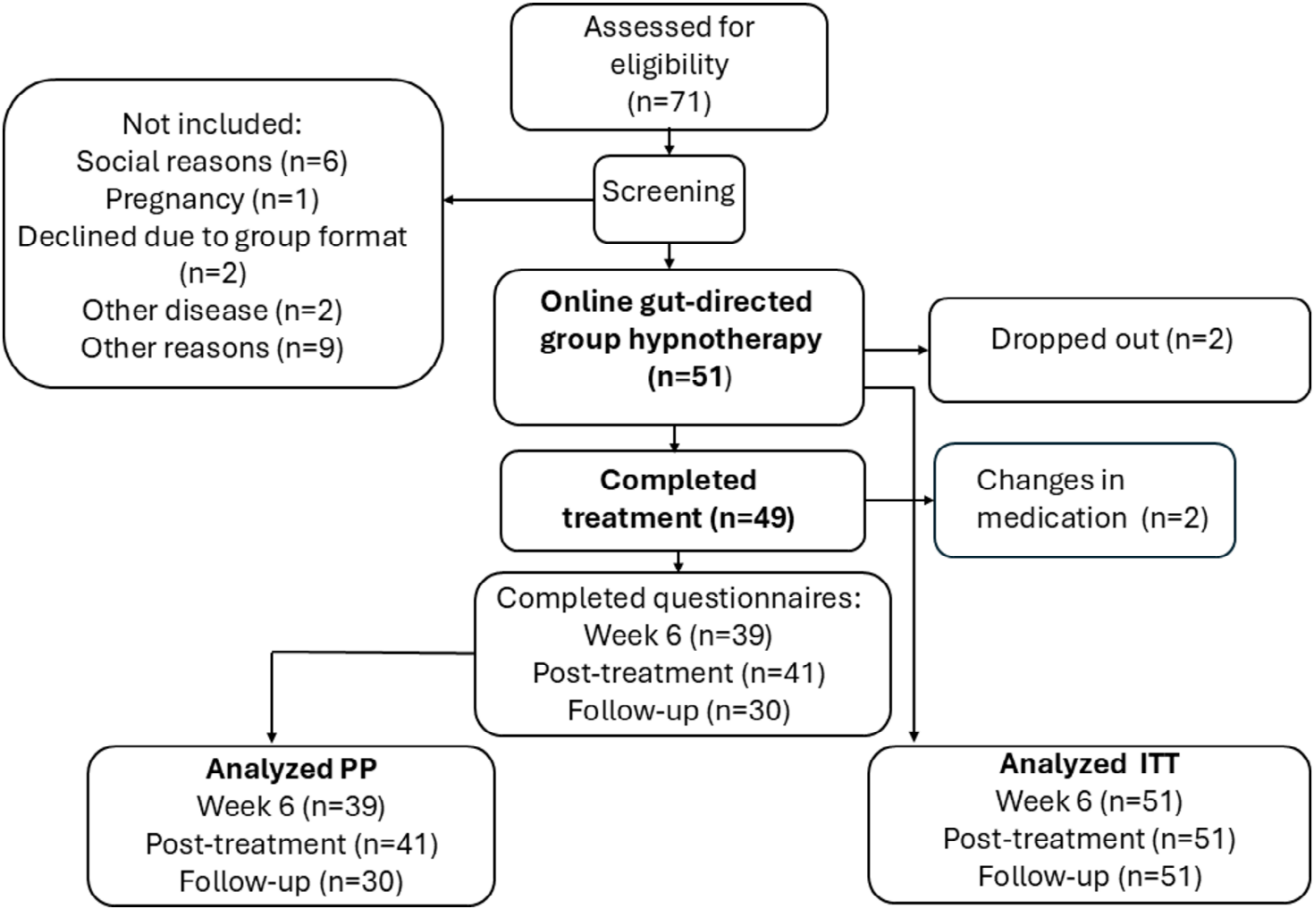
Flowchart demonstrating the number of patients during different phases of the online hypnotherapy study.

Seventy‐one patients with treatment‐refractory IBS were assessed for study participation. After the screening process, 51 patients (mean age 38 [range: 19–74]years, 42 females) were included in the study and received the online group hypnotherapy treatment. A common reason for exclusion was recent changes in medications that could affect GI symptoms. The median length of history duration of IBS symptoms was 17 (range: 2–57) years. The majority of the patients reported moderate or severe IBS symptoms, and there was a slight predominance of IBS with diarrhea (Table 2). Two patients terminated the treatment prematurely, one due to pregnancy and the other reported a lack of time for the treatment. Two patients made changes in their medication during the intervention. For these patients, the most recent symptom assessments prior to the change in medication were carried forward in the ITT analysis. Out of all 49 patients who received at least seven of the eight sessions of gut‐directed hypnotherapy, 41 patients completed the symptom assessments posttreatment and were included in the per‐protocol analyses. No patients reported any adverse reactions during treatment. The flow chart for the online group hypnotherapy is shown in Figure 2.

**TABLE 2:** Baseline characteristics of online group hypnotherapy patients (*N* = 51).

Females, *n* (%)	42 (82)
Age, mean (range)	38 (19–74)
Years with IBS, mean (range)	17 (2–57)
IBS subtype, *n* (%)	
IBS with diarrhea	24 (47)
IBS with constipation	14 (27)
Mixed IBS	13 (25)
IBS‐SSS, median (IQR)	304 (225–385)
Mild (score ≤ 175), *n* (%)	2 (4)
Moderate (score 175–300), *n* (%)	23 (45)
Severe (score ≥ 300), *n* (%)	26 (51)
Extracolonic score, median (IQR)	201 (165–233)
VSI, median (IQR)	54 (40–63)
HADS anxiety, median (IQR)	11 (7–15)
HADS depression, median (IQR)	6 (4–8)

Abbreviations: IBS‐SSS, IBS severity scoring system; HADS, hospital anxiety and depression scale; VSI, visceral sensitivity index.

### 3.2 IBS Symptoms

**FIGURE 3:**
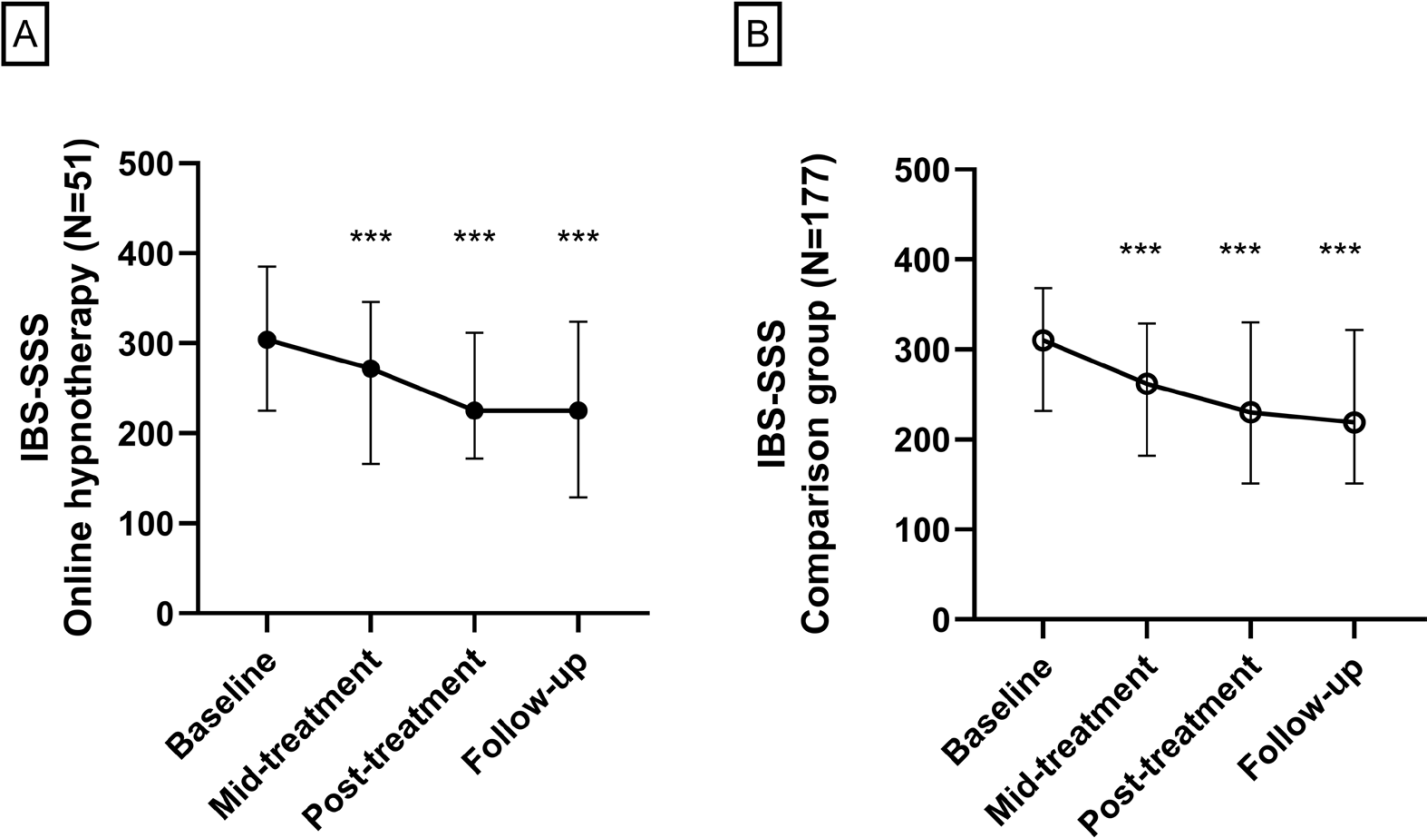
IBS symptom severity for (A) online group hypnotherapy patients, *N* = 51 (IBS‐SSS; median, IQR), at baseline, mid‐treatment, posttreatment and follow‐up and for (B) patients receiving group hypnotherapy on‐site. ***p < 0.001 versus baseline.

The IBS symptoms (overall IBS‐SSS score) improved after hypnotherapy (*p* < 0.001), and this improvement was sustained at follow‐up assessments 6 months after treatment initiation (Table 3). A significant positive treatment effect was seen already halfway through the treatment (*p* < 0.001, Figure 3A). Significant positive changes following hypnotherapy were seen in four of the individual components of the IBS‐SSS; abdominal pain severity, abdominal pain frequency, bowel habit dissatisfaction, and life interference. (Table S1). A total of 53% (27 out of 51) of the patients who underwent the intervention fulfilled the responder criterion at posttreatment, and at follow‐up, 57% (29 out of 51) fulfilled the responder criterion. The proportion of patients classified with severe IBS was reduced from 51% to 29% posttreatment. The proportion of patients who had mild IBS increased from 4% at baseline to 24% posttreatment. The changes in IBS severity classification seen at the posttreatment assessment were sustained at follow‐up (Figure S1).

**TABLE 3:** IBS symptoms, extracolonic, and psychological symptoms for the online group hypnotherapy patients.

	Baseline	Posttreatment	6‐month follow‐up
IBS‐SSS score	304 (225–385)	225 (172–312)^c^	225 (129–324)^c^
IBS‐SSS extracolonic score	201 (165–233)	154 (89–201)^c^	155 (103–206)^c^
VSI score	54 (40–63)	45 (28–56)^c^	37 (23–50)^c^
HADS anxiety score	11 (7–15)	9 (5–13)^a^	8.5 (6–11)^a^
HADS depression score	6 (4–8)	4 (2–7)^b^	4 (2–7)^b^

*Note:* ITT data for IBS‐SSS score (*N* = 51), Per‐Protocol data for other variables (*N* = 30). Median (IQR).

Abbreviations: IBS‐SSS, IBS severity scoring system; HADS, hospital anxiety and depression scale; VSI, visceral sensitivity index.

^a^ *p* < 0.05 versus baseline.

^b^ *p* < 0.01 versus baseline.

^c^ *p* < 0.001 versus baseline.

### 3.3 Secondary Endpoints

Improvements in the severity of GI‐specific and general anxiety, depression, as well as extracolonic symptoms were seen posttreatment, with sustained effects at follow‐up (Table 3). Statistically significant improvements were also noted regarding quality of life in seven of the nine assessed domains posttreatment (Table 4). The positive changes in quality of life were sustained at follow‐up. Patient ratings of the usability of the software application were high, with a median score of 95 (IQR: 87–100). The median score of the Client Satisfaction Questionnaire was 25 (IQR: 21–30), which is a relatively high score and in line with reported outcomes in other recent studies where the questionnaire has been used for evaluation of different mental health care services given both on‐site and online [32, 33]. When asked about treatment modality preference, most of the participants (35 out of 48, 73%) stated that they would have preferred to receive hypnotherapy remotely and not on‐site. The main reasons for this were that it was practical, easy to access, and saved time. Patients also stated that it was easy to relax at home, and that they thought it would be harder to relax in a hospital environment with other participants in the same room. A smaller subgroup stated that they would have chosen hypnotherapy on‐site (7 out of 48, 14.5%), mainly because they thought that it could be more effective than remote hypnotherapy. Another reason was that it could be easier to connect with other group members if you met them in‐person. Six patients (12.5%) did not state any preference among the two treatment modalities. It was common among these six participants to say that the two modalities probably had both positive and negative aspects. There were some technical problems during the hypnotherapy sessions, such as audio issues or disconnections from the video session, but the problems occurred less frequently over time due to technical improvements of the telehealth application during the study. A small number of patients (5 out of 48) brought this up as a negative aspect of online hypnotherapy during the treatment evaluation, although it was not described as a dealbreaker that made them choose in‐person treatment instead (Table 5). Individuals classified as nonresponders after hypnotherapy did not differ from responders in their assessments of system usability (SUS median (IQR): 96.2 (88–100) vs. 92.5 (87.5–100), *p* = 0.52), treatment satisfaction. (CSQ‐8 median (IQR): 25 (21.5–27.8) vs. 27 (20–30), *p* = 0.19), or their stated treatment preference (online preference 15/23 vs. 20/25, *p* = 0.095).

**TABLE 4:** Quality of life for the online group hypnotherapy patients.

	Baseline	Posttreatment	Follow‐up
Emotional functioning	38 (25–50)	50 (25–69)^c^	56 (34–75)^b^
Mental health	60 (40–80)	70 (50–85)^c^	80 (58–85)^c^
Sleep	67 (50–92)	83 (67–100)^c^	83 (63–100)^a^
Energy	50 (25–63)	50 (38–75)^b^	75 (38–75)^b^
Physical functioning	75 (50–92)	75 (58–92)	58 (83–100)
Diet	40 (53–68)	53 (67–80)^c^	73 (47–80)^b^
Social role	47 (25–70)	63 (44–81)^c^	63 (44–81)^a^
Physical role	44 (19–63)	50 (31–75)^a^	50 (31–88)^a^
Sexual relations	58 (25–92)	75 (54–100)	92 (50–100)

*Note:* Per‐protocol data (*N* = 30), median (IQR).

^a^ *p* < 0.05 versus baseline.

^b^ *p* < 0.01 versus baseline.

^c^ *p* < 0.001 versus baseline.

**TABLE 5:** The views of the study patients on online versus in‐person hypnotherapy.

Online	In‐person
Pros	
Practical	Maybe more effective
Time saving	Easy to connect with other group members
Easy to relax	
Accessible	
Cons	
Technical problems	Time‐consuming
Maybe less effective	Difficult to relax in the hospital
Difficulties connecting with group members	Difficult to access due to distance to hospital or incapacitating symptoms

### 3.4 Comparison of Online Group Hypnotherapy With In‐Person Group Hypnotherapy

A total of 177 patients (mean age: 39, 18–70 years, 130 females) were included in the comparison group; of these, 58 had participated in the RCT [10], and the other 119 had been treated clinically at the unit [11]. Of all the 177 patients included, 158 completed the hypnotherapy program (Figure S2). Baseline characteristics were similar to the group receiving online group hypnotherapy (Table S2).

Improvements in IBS symptoms seen in the comparison group were comparable with those in the online hypnotherapy group (Figure 3B and Table S2). The proportion of responders was 55% (98 out of 177) after on‐site group hypnotherapy treatment, and 57% (101 out of 177) at follow‐up, which is comparable to the results with the online group hypnotherapy (53% and 57%, respectively). The proportion of patients with severe IBS at baseline was similar in the two groups (54% and 50% in the on‐site and online hypnotherapy groups, respectively) and was reduced in both groups after treatment (32% on‐site vs. 29% online). Similarly, extracolonic symptoms, GI‐specific and general anxiety, and depression all improved significantly and to a comparable extent after treatment in both groups, with significant improvements sustained in all assessments at follow‐up except for depression (Table S3).

## 4 Discussion

This study aimed to test whether gut‐directed hypnotherapy given to groups of patients via live video conferencing is feasible and effective. As of now, this is the only study where group and online delivery of gut‐directed hypnotherapy have been combined. Our study clearly shows that online group hypnotherapy is feasible and has efficacy comparable to the same treatment delivered on‐site. Hence, this new treatment strategy has the potential to increase accessibility considerably, without a reduction in symptom relief.

Most patients who received the online treatment preferred this modality over in‐person hypnotherapy at the hospital. One of the most common positive comments about online hypnotherapy among the participants was that it was easier to relax in one's own home compared to in a hospital group setting. Despite some minor technical problems during the online sessions, patients thought that the video conferencing application was user‐friendly and well‐functioning. Similarly, previous findings from the Manchester group indicate that patients receiving individual hypnotherapy remotely reported minor technical problems but were generally satisfied with the functioning of the telehealth platform [34]. The median score of the System Usability Scale (SUS) was high, which indicates that the study participants were satisfied with how the software application worked. The feedback regarding treatment preference shows that the positive aspects of the treatment modality clearly outweigh any drawbacks. Given the fact that this is a relatively time‐consuming treatment, a remote delivery mode could save valuable time for patients and enable accessibility to treatment for those who would not have had the possibility to participate otherwise. The median outcome score from the Client Satisfaction Questionnaire in this study was within the mean range compared to where mental health services have been provided face‐to‐face at outpatient and inpatient clinics [31]. This further confirms that the treatment modality is acceptable for patients.

Patients showed significant improvement in overall IBS severity score at mid‐treatment, and this positive change was even more distinct after the hypnotherapy program. The changes were sustained at follow‐up 6 months after treatment initiation. This is in line with symptom improvements noted for the comparison group who received group hypnotherapy on‐site. The proportion of successfully treated online group hypnotherapy patients after treatment (53%) was comparable to the responder rate for the comparison group (55%). At follow‐up, the responder rates for the two groups were the same (57%), implying that remote delivery is as efficacious as when hypnotherapy is given on‐site. However, it should be stressed that formal comparisons within a randomized controlled trial should ideally be done to run proper direct statistical comparisons between the two delivery strategies. Notably, differences in responder rates between group and individual hypnotherapy have been reported in our previous studies [10, 11], with a higher proportion of responders observed for individual delivery (69% and 64.3%). Improvements were also seen regarding the secondary endpoints, that is GI‐specific and general anxiety, and depressive symptoms, as well as extracolonic symptoms and quality of life, which further confirm that online group hypnotherapy seems to be as beneficial for IBS patients as conventional group hypnotherapy delivered on‐site. When remote hypnotherapy has been tested in previous studies, slightly lower effectiveness compared to conventional hypnotherapy on‐site has been reported. The response rate using hypnotherapy audio tapes did not reach the same high levels as for the therapist‐delivered hypnotherapy [12]. In another study, individual hypnotherapy delivered on a video platform was reported to be effective, but with a tendency toward smaller symptom reduction compared to therapist‐delivered hypnotherapy on‐site [17]. Smartphone app‐delivered hypnotherapy, provided through prerecorded sessions, could be an attractive alternative to conventional, therapist‐delivered hospital‐based hypnotherapy, with the potential to reach more patients. However, adherence to treatment seems to be a problem. Peters et al. reported that only 18% of those who purchased the hypnotherapy app completed all sessions. For the program completers, however, hypnotherapy was effective, and 64% reached the responder criteria after treatment [16]. In our study, there were no clear differences in effectiveness between the online hypnotherapy group and the comparison group. The proportions of successfully treated patients were similar in the two groups posttreatment, and the same was seen at follow‐up. One possible reason for the similar results for the two groups in this study could be that, despite the remote delivery form, the treatment was still provided live by a health care provider, compared to several other remote delivery forms, such as audio recordings or through an app where you listen to a prerecorded voice. Prerecorded sessions might be perceived as less personal or effective for the patient. Hypnotherapy via audio recordings could also add more responsibility to the patient to adhere to treatment without having any booked appointments or hypnotherapy sessions with a therapist. The considerably lower dropout rates observed in our study, compared with app‐based, non‐therapist‐delivered hypnotherapy, may partly be explained by this.

This study has some limitations. It is not a randomized controlled trial, and no non‐inferiority analyses or statistical comparisons were performed between the online treatment group and the comparison group. However, the purpose of the study was to test feasibility of the new delivery format and to assess if treatment outcomes were comparable with on‐site treatment. Therefore, a pilot study was considered the most appropriate first step. Although the outcomes are very promising, online group hypnotherapy would need to be tested in a larger cohort, preferably at different treatment centers, and in the form of a randomized controlled trial to establish reproducibility and efficacy. There is also a need for long‐term evaluations to see if the effects are lasting as long as for in‐person hypnotherapy [8, 11, 24, 35]. Furthermore, the symptom assessments of the online hypnotherapy group were performed digitally, while the comparison group assessed symptoms via paper questionnaires. In addition to this, the question of treatment preference at posttreatment could only be answered in a hypothetical manner since the patients only had experience from online hypnotherapy and never had tested hypnotherapy on‐site. Nonetheless, we believe that the answers provided valuable insights into how participants have experienced this new way of delivering hypnotherapy.

One strength of this study lies in the standardized and uniform way (scripted protocol) that gut‐directed hypnotherapy was given to all patients. The North Carolina protocol is widely used to treat IBS patients and therefore the outcome of this study can be applicable to several other centers using this protocol. Another strength is the robust data from 177 patients in the comparison group, and the fact that these patients received exactly the same treatment as the study participants, except for the remote delivery. The comparison group was similar to the pilot study group regarding the baseline characteristics, which enabled reliable evaluation and comparison between groups. Furthermore, the sample size for this pilot study group is relatively large, which increases the reliability of the outcomes.

Individual hypnotherapy has been associated with a somewhat higher proportion of responders compared to group‐based delivery [10, 11]. Ideally, this modality could be offered to patients most likely to benefit, perhaps those with more severe symptoms or greater comorbidity, while also considering patient preferences. However, such an approach may be difficult to implement in routine clinical practice due to limited resources, and there is currently limited evidence to guide the selection of patients for individual versus group treatment. Similar uncertainties apply to the choice between remote and on‐site delivery. One study reported that IBS patients who, after online hypnotherapy, indicated a preference for in‐person treatment, were more likely to have a poorer treatment response [34], a finding not observed in our study but potentially relevant for treatment selection. Additionally, difficulties relaxing at home due to in‐home distractions have been reported as a limitation of remote hypnotherapy [20, 34]. Although this was not identified as a concern in our cohort, it may be relevant for patients who anticipate such challenges at home. As technology advances, it is becoming more common to offer a variety of mental health interventions remotely. Brain–gut behavioral therapies such as gut‐directed hypnotherapy seem to be well suited for remote delivery by using digital solutions. To conclude, this pilot study shows that online gut‐directed hypnotherapy delivered in a group format is feasible, efficacious, and often preferred over in‐person hypnotherapy. Hence, this could be an effective alternative to conventional hypnotherapy delivered on‐site, and the delivery format has the potential to increase accessibility.

## Author Contributions

**Jenny Lövdahl:** study concept and design, administration of the hypnotherapy treatment, acquisition of data, analysis and interpretation of data, drafting of the manuscript, critical revision of the manuscript for important intellectual content, obtained funding. **Hans Törnblom:** study concept and design, referring patients to the study, critical revision of the manuscript for important intellectual content. **Gisela Ringström:** critical revision of the manuscript for important intellectual content. **Olafur S. Palsson:** author of the hypnotherapy protocol, critical revision of the manuscript for important intellectual content. **Magnus Simrén:** study concept and design, referring patients to the study, analysis and interpretation of data, critical revision of the manuscript for important intellectual content, study supervision, obtained funding. All authors have approved the final version of the article, including the authorship list.

## Funding

This work was supported by Swedish governmental funding of clinical research (ALFGBG‐726561, ALFGBG‐965173, ALFGBG‐722331, ALFGBG‐983998).The Swedish Research Council (2018‐02566, 2021‐00947). The Healthcare Committee, Region Västra Götaland (VGFOUREG 855971, VGFOUREG 930214).

## Conflicts of Interest

The authors declare no conflicts of interest.

## Disclosure

The authors have nothing to report.

## Supporting information


**Figure S1:** Severity online hypnotherapy.

**Figure S2:** Flow chart comp group.

**Table S1:** Change in overall and individual components of the IBS‐SSS after treatment for online group hypnotherapy patients.
**Table S2:** Baseline characteristics of the comparison group (*N* = 177).
**Table S3:** IBS‐symptoms, extracolonic and psychological symptoms for the comparison group.

## Data Availability

The data that support the findings of this study are available from the corresponding author upon reasonable request.
