# Supplementary figures and images for: Online Group Hypnotherapy for Irritable Bowel Syndrome—a Pilot Study
Source: Neurogastroenterol Motil. 2026 Apr 29;38:e70328. doi: 10.1111/nmo.70328 (PMC13129414; doi:10.1111/nmo.70328)

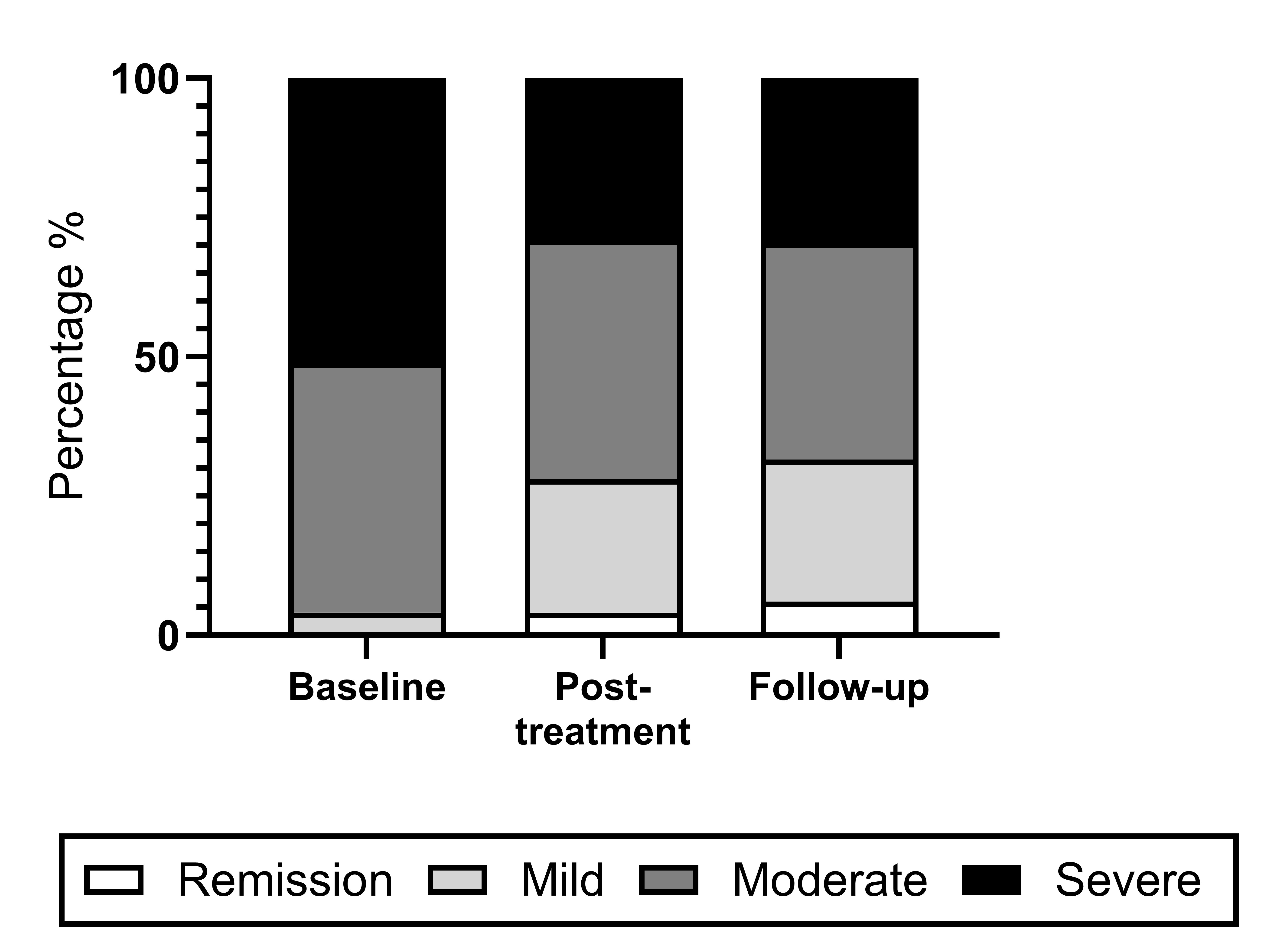

Supplement: Supplementary file 1 — Figure S1: Severity online hypnotherapy. [file NMO-38-e70328-s002.png]

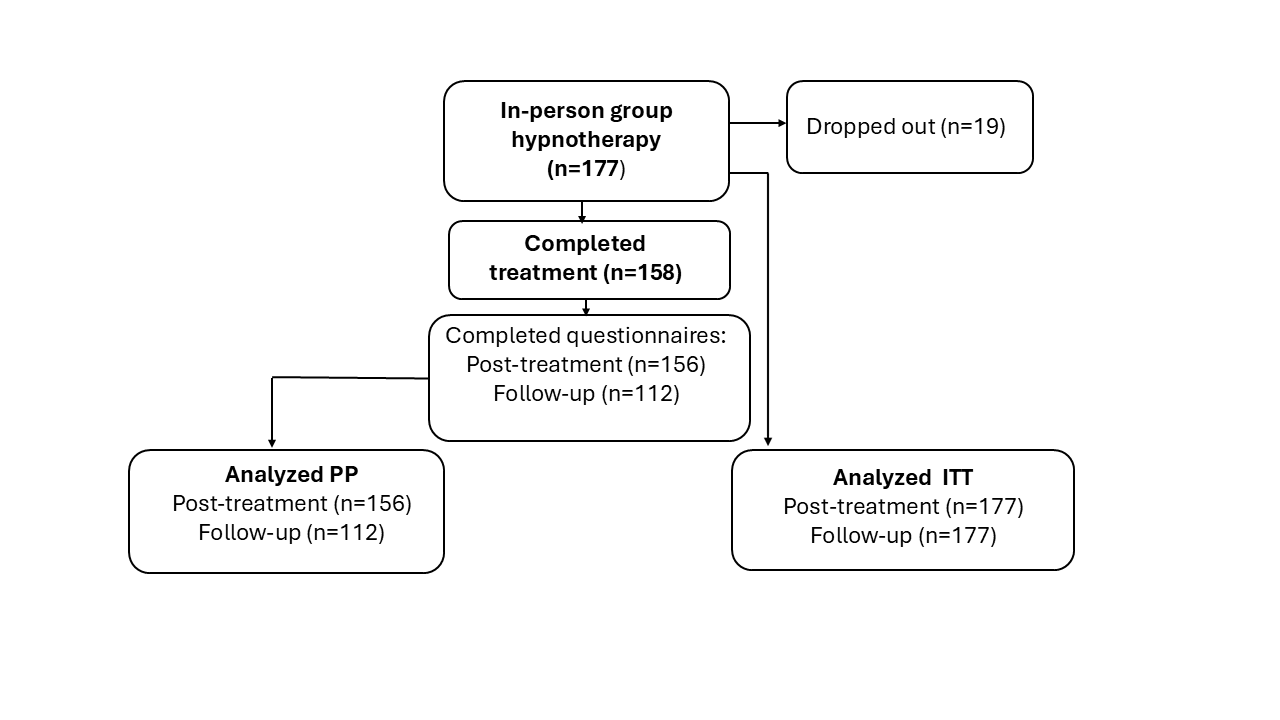

Supplement: Supplementary file 2 — Figure S2: Flow chart comp group. [file NMO-38-e70328-s001.png]
