# Supplementary material for: Online Group Hypnotherapy for Irritable Bowel Syndrome—a Pilot Study
Source: Neurogastroenterol Motil. 2026 Apr 29;38:e70328. doi: 10.1111/nmo.70328 (PMC13129414; doi:10.1111/nmo.70328)
Supplement: Supplementary file 3 — Table S1: Change in overall and individual components of the IBS‐SSS after treatment for online group hypnotherapy patients. Table S2: Baseline characteristics of the comparison group (N = 177). Table S3: IBS‐symptoms, extracolonic and psychological symptoms for the comparison group. [file NMO-38-e70328-s003.docx]

| SUPPORTING INFORMATION  Suppl. Table 1. Change in overall and individual components of the IBS-SSS after treatment for online group hypnotherapy patients | | | |
| --- | --- | --- | --- |
| Variable | Baseline | Post-treatment | p-value |
| Overall IBS-SSS score | 304 (225‒385) | 225 (172‒312) | <0.001 |
| Pain severity | 47 (17‒57) | 29 (0‒55) | <0.05 |
| Pain frequency | 70 (20‒90) | 20 (0‒60) | <0.001 |
| Abdominal bloating | 49 (30‒76) | 43 (0‒68) | 0.287 |
| Bowel habit dissatisfaction | 77 (68‒97) | 58 (37‒79) | <0.05 |
| Life interference | 91 (70‒100) | 67 (49‒88) | <0.001 |

Abbreviations: IBS-SSS, IBS Severity Scoring System. ITT data (N=51). Median (IQR).

Suppl. Table 2. Baseline characteristics of the comparison group (N=177)

| Females, n (%) | 130 (73) |
| --- | --- |
| Age, mean (range) | 39 (18‒70) |
|  |  |
| IBS subtype, n (%) |  |
| IBS with diarrhea | 82 (46) |
| IBS with constipation | 41 (23) |
| Mixed IBS | 54 (31) |
|  |  |
| IBS-SSS, median (IQR) | 310 (232‒368) |
| Mild (score ≤175), n (%) | 17 (10) |
| Moderate (score 175-300), n (%) | 64 (36) |
| Severe (score ≥300), n (%) | 96 (54) |
|  |  |
| Extracolonic score, median (IQR) | 182 (123‒247) |
| VSI, median (IQR) | 46 (34‒58) |
| HADS anxiety, median (IQR) | 9 (6‒13) |
| HADS depression, median (IQR) | 6 (3‒8) |
|  |  |

Abbreviations: IBS-SSS, IBS Severity Scoring System; VSI: Visceral Sensitivity Index; HADS:

Hospital Anxiety and Depression Scale.

| Suppl. Table 3. IBS-symptoms, extracolonic and psychological symptoms for the comparison group | | | | | | | | | |  |
| --- | --- | --- | --- | --- | --- | --- | --- | --- | --- | --- |
|  |  | Baseline |  | Post-treatment | |  | Follow-up | | | |
| IBS-SSS score |  | 310 (232‒368) |  | 230 (151‒330) *** |  |  | 219 (151‒322) *** | | | |
| IBS-SSS extracolonic score | | 182 (123‒247) |  | 126 (63‒211) *** |  |  | 126 (73‒196) *** | | | |
| VSI score |  | 46 (34‒58) |  | 37 (25‒50) *** |  |  | 34 (22‒49) *** | | | |
| HADS anxiety score |  | 9 (6‒13) |  | 8 (4‒12) *** |  |  | 8 (5‒12) ** | | | |
| HADS depression score |  | 6 (3‒8) |  | 4.5 (2‒8) * |  |  | 6 (2‒8) | | | |
|  |  |  |  |  |  |  | |  |  | |

Note: ITT data for IBS-SSS score (N=177), Per Protocol data for other variables (N=156). Median (IQR).

Abbreviations: IBS-SSS, IBS Severity Scoring System; VSI, Visceral Sensitivity Index; HADS, Hospital Anxiety and Depression Scale.

*p< 0.05 vs baseline; **p< 0.01 vs baseline; ***p< 0.001 vs baseline
